# Exposure to Mosquito Coil Smoke May be a Risk Factor for Lung Cancer in Taiwan

**DOI:** 10.2188/jea.18.19

**Published:** 2008-02-28

**Authors:** Shu-Chen Chen, Ruey-Hong Wong, Li-Jie Shiu, Ming-Chih Chiou, Huei Lee

**Affiliations:** 1 Institute of Medicine, Chung Shan Medical University; 2 Nursing School, Central Taiwan University of Science and Technology; 3 Department of Public Health, Chung Shan Medical University; 4 Department of Surgery, Chung Shan Medical University and Hospital; 5 Institute of Medical & Molecular Toxicology, Chung Shan Medical University

**Keywords:** Air Pollutants, Lung Neoplasms, Mosquito Coil, Smoke

## Abstract

**Background:**

About 50% of lung cancer deaths in Taiwan are not related to cigarette smoking. Environmental exposure may play a role in lung cancer risk. Taiwanese households frequently burn mosquito coil at home to repel mosquitoes. The aim of this hospital-based case-control study was to determine whether exposure to mosquito coil smoke is a risk for lung cancer.

**Methods:**

Questionnaires were administered to 147 primary lung cancer patients and 400 potential controls to ascertain demographic data, occupation, lifestyle data, indoor environmental exposures (including habits of cigarette smoking, cooking methods, incense burning at home, and exposure to mosquito coil smoke ), as well as family history of cancer and detailed medical history.

**Results:**

Mosquito coil smoke exposure was more frequent in lung cancer patients than controls (38.1% vs.17.8%; p<0.01). Risk of lung cancer was significantly higher in frequent burners of mosquito coils (more than 3 times [days] per week) than nonburners (adjusted odds ratio = 3.78; 95% confidence interval: 1.55-6.90). Those who seldom burned mosquito coils (less than 3 times per week) also had a significantly higher risk of lung cancer (adjusted odds ratio = 2.67; 95% confidence interval: 1.60-4.50).

**Conclusion:**

Exposure to mosquito coil smoke may be a risk factor for development of lung cancer.

## INTRODUCTION

Lung cancer is a major cause of cancer mortality globally.^1^ In Taiwan, lung cancer is also the leading cause of cancer death.^2^^,^^3^ While cigarette smoking is the most important cause of lung cancer in both men and women in the Western world, it is responsible for only about 50% of lung cancer death in Taiwan.^2^^,^^3^ Thus, other factors contributed to lung cancer development, e.g. passive smoking.^4^ Moreover, previous studies indicated that environmental exposures to indoor and outdoor air pollutants such as cooking oil fumes,^5^^,^^6^ radon,^7^ and asbestos,^8^ were associated with elevated lung cancer risk.

People in residences are often protected from nuisance and disease-bearing mosquitoes by insecticides or smoke generated from burning mosquito coils. Mosquito coils are frequently burned indoors in Asia and to a limited extent in other parts of the world, including the United States.^9^ In 1996, a World Health Organization (WHO) report estimated the worldwide annual consumption of mosquito coils to be approximately 29 billion pieces.^10^ The prevalence of families that burn mosquito coils in Taiwanese is approximately 45%.^11^ The major active ingredients of the mosquito coil are pyrethrins, accounting for about 0.3-0.4% of coil mass.^12^ The bulk of mosquito coils consists of plant-based materials, such as wood powder, coconut shell powder, joss powder, binders, dyes, oxidants (e.g., nitrates), and other additives making controlled smoldering possible during their use of approximately 8 hours. When a mosquito coil is burned, the insecticides evaporate with the smoke, which immobilizes the mosquito and prevents it from entering the room. The combustion of the remaining materials generates large amounts of submicrometer particles and gaseous pollutants. These submicrometer particles may reach the lower respiratory tract and be coated with a wide range of organic compounds such as polycyclic aromatic hydrocarbons. Moreover, burning one mosquito coil releases the same amount of particulate matter (PM2.5) as burning 75-137 cigarettes. Also, the emission of formaldehyde from burning one coil can be as high as that released from burning 51 cigarettes.^13^ Furthermore, long-term exposure to mosquito coil smoke (MCS) might induce asthma and persistent wheeze in children.^14^^,^^15^

Frequently, mosquito coils also contain octachlorodipropyl ether (S-2, S-421) as a synergist or active ingredient.^16^ S-2 may be volatized from burning mosquito coils. In particular, hydrogen chloride and formaldehyde are formed from combustion of S-2.^17^ Further, hydrogen chloride and formaldehyde react in smoky air to form bis(chloromethyl)ether.^18^ The degradation of S-2 to bis(chloromethyl)ether during the burning of mosquito coils is of particular concern^10^ because bis(chloromethyl)ether is an extremely potent lung carcinogen.^19^^,^^20^

This hospital-based case-control study aims to determine whether exposure to MCS is a risk factor for lung cancer development in Taiwan.

## METHODS

### Selection of Case and Controls

Between July 2002 and February 2004, subjects were recruited from three medical centers in the Taichung metropolitan area (central Taiwan). Most subjects lived in central Taiwan, while some lived in other areas and were referred from other hospitals. Primary lung cancer (International Classification of Diseases, 9th revision; code 162) was newly diagnosed and histopathologically confirmed by experienced pathologists or chest specialists. Among 148 patients with newly identified lung cancer, one was too ill to participate and the other 147 all agreed to participate in the study. During the same period, hospital-based controls were recruited from the Ophthalmology and Family Medicine Departments in the same medical centers, including patients with cataracts, glaucoma, corneal opacity, retinal detachment, as well as some patients who came in for a general health check-up. These controls were also selected from the same geographic areas as the lung cancer patients. Sixteen patients with other types of cancer were also excluded. A total of 889 hospital controls were available, and 400 control subjects (eye problems, 57%; physical check-up, 43%) agreed to participate in the study. Primary reason for the low response rate of our controls was that most subjects were unwilling to take the time for interview.

### Epidemiologic Data

After selection of cases and controls, a trained professional interviewer performed a face-to-face interview to collect data in an orally administrated questionnaire at the hospital within the study period. An informed consent from each subject was also obtained. This interviewer was trained to treat patients and controls in a similar manner and was masked to the participants’ group assignment. The average interview time was 37 and 33 minites, respectively, for patients and controls. To minimize possible biases in quantitative data resulting from incorrect recall, a structured questionnaire was used to collect categorical information. The structured questionnaire covered demographic characteristics, occupation, lifestyle, and indoor environmental exposures (including habits of cigarette smoking, and cooking methods, incense burning at home, and exposure to MCS), as well as family history of cancer and personal medical history (such as history of tuberculosis). Family history of cancer was defined as the presence of any cancer within first-degree relatives. Information obtained from the questionnaire was used to assign industry and job codes according to the International Standard Classification of Occupations (ISCO, Revised Edition 1968)^21^ and the Statistical Classification of Economic Activities in the European Community (NACE Rev.1).^22^ Subsequently, occupations were classified into one of four categories: administrative, hazardous industry, farmer, and housewife, and a person’s lifetime occupation was taken to be the job they held the longest. Hazardous occupations/industries were defined as those associated with exposures to known or suspected carcinogens of lung cancer. These included mining and quarrying, chemical/pesticide production, asbestos production, metals manufacturing, shipbuilding, motor vehicle manufacturing and repair, railroad equipment manufacturing, gas production, construction, rubber industry, leather industry, printing, and wood and wood products manufacturing and transport. The subject’s active smoking history included the number of cigarettes smoked daily and the duration of the subject’s smoking habit. A regular smoker was defined as those who smoked one or more cigarettes per day for at least one year. Information about patients and controls exposed to environmental tobacco smoke was also collected. Questions on passive smoking included “have you lived or worked with any regular smokers?” Those who lived or worked with a smoker(s) and were exposed to tobacco smoke were considered to be passive smokers. Past domestic exposure to smoke during cooking was also evaluated. Subjects were asked about the frequency of using various cooking methods, particularly stir-frying. In addition, exposure to incense was defined as regular exposure to incense burning in an enclosed space. Exposure to MCS was also defined as regular exposure to MCS at home. To ascertain MCS exposure, subjects were asked "in the past, did you burn mosquito coils at home against mosquitoes?" Once mosquito coil burning was established, the frequency of mosquito coil burning was ascertained from the five possible answers to the question "how often do you burn mosquito coils (per day or per week)?," i.e., every day (more than one time per day), three to four times per week, one to two times per week, one to two times per month, and seldom.

### Statistical Analysis

The age, sex, marital status, occupation, smoking status, exposure to cooking oil fumes, exposure to incense, exposure to MCS, tuberculosis history, and family history of cancer of the patient and control groups were compared using the *t* test for continuous variables and chi-square test for discrete variables. Subsequently, a multiple unconditional logistic model was employed to obtain the adjusted odds ratio (OR) and 95% confidence interval (CI) for selected variables after adjusting for the effect of potential confounding factors. The joint effect of MCS exposure and cigarette smoking was also estimated by the synergy index,^23^ which is the ratio between the observed excess risk in those with exposures to two risk factors (OR_11_ - OR_00_) and the excess risk predicted under simple additivity (the sum of two excess risks with only exposure to two risk factors, i.e., [OR_10_ - OR_00_]+[OR_01_ - OR_00_]). A confidence interval was also calculated using the delta method.^24^ A synergy index greater than 1 indicated the synergistic effect of the two risk factors on lung cancer development. We used SAS^®^ statistical software, version 9.1 (SAS Institute Inc., Cary, NC, USA) for all analyses. All p-values were calculated using two-tailed statistical tests, and the criterion for significance was set at p<0.05.

## RESULTS

Of the 147 patients with histologically confirmed primary lung cancer, 91 (61.9%) had adenocarcinoma and 30 (20.4%) squamous cell carcinoma. The demographic information and environmental exposures of all subjects are summarized in Table 1. The mean age of patients and controls was 63.7 ± 12.7 (standard deviation [SD]) and 56.3±11.5 years (p<0.01, t test), respectively. Sex did not significantly differ between the two groups. The percent with single marital status (including single, separated, divorced, or widowed) was significantly higher in patients than controls (15.6% vs. 8.0%; p<0.01, chi-square test). In addition, patients were more likely than controls to work in hazardous industry environments (35.4% vs. 24.8%; p<0.01).

**Table 1.** Distribution of selected characteristics of study cases of lung cancers and controls.

	Patients	Controls	All
	(n=147)	(n=400)	(n=547)
Age	63.7 ± 12.7^*,^^†^	56.3 ± 11.5	58.3 ± 12.3
Sex			
Male	80 (54.4%)^‡^	226 (56.5%)	306 (55.9%)
Female	67 (45.6%)	174 (43.5%)	241 (44.1%)
Marital status			
Single	23 (15.6%)^†^	32 ( 8.0%)	55 (10.0%)
Couple	124 (84.4%)	368 (92.0%)	492 (90.0%)
Occupations			
Hazardous industry	52 (35.4%)^†^	99 (24.8%)	151 (27.6%)
Administration	46 (31.3%)	205 (51.2%)	251 (45.9%)
Farmer	36 (24.5%)	64 (16.0%)	100 (18.3%)
House wife	13 ( 8.8%)	32 ( 8.0%)	45 ( 8.2%)
Active smoking			
Yes	68 (46.3%)^†^	107 (26.8%)	175 (32.0%)
No	79 (53.7%)	293 (73.2%)	372 (68.0%)
Packing year smoked	3.9 ± 3.4^*,^^†^	3.0 ± 3.6	3.3 ± 3.1
Passive smoking			
Yes	42 (28.6%)	94 (23.5%)	136 (24.9%)
No	105 (71.4%)	306 (76.5%)	411 (75.1%)
Exposure to cooking oil fume			
Yes	93 (63.3%)	226 (56.5%)	319 (58.3%)
No	54 (36.7%)	174 (43.5%)	228 (41.7%)
Exposure to incense			
Yes	90 (61.2%)	242 (60.5%)	332 (60.7%)
No	57 (38.8%)	158 (39.5%)	215 (39.3%)
Exposure to mosquito coil smoke			
Yes	56 (38.1%)^†^	71 (17.8%)	127 (23.2%)
No	91 (61.9%)	329 (82.3%)	420 (76.8%)
Tuberculosis history			
Yes	16 (10.9%)^†^	17 ( 4.3%)	33 ( 6.0%)
No	131 (89.1%)	383 (95.7%)	514 (94.0%)
Family history of cancer			
Yes	24 (16.3%)	50 (12.5%)	74 (13.5%)
No	123 (83.7%)	350 (87.5%)	473 (86.5%)
Histological type			
Adenocarcinoma	91 (61.9%)		
Squamous cell carcinoma	30 (20.4%)		
Large cell carcinoma	6 ( 4.1%)		
Small cell carcinoma	20 (13.6%)		

* : Mean ± standard deviation

† : p<0.01; Chi-square test or t-test

‡ : Number (%)

To verify whether exposure to MCS was a risk factor for lung cancer, the well-known risk factors for lung cancer in Taiwan (including cigarette smoking, passive smoking, exposure to cooking oil fumes, exposure to incense smoke, tuberculosis infection as well as family history of cancer) were also included in our statistical analysis. As expected, the proportion of active cigarette smokers, those exposed to MCS, and those with tuberculosis infections, and the number of pack-years of smoking was higher among lung cancer patients than controls.

Furthermore, we tested whether there was an association between lung cancer risk and frequency of mosquito coil use (Table 2). The risk of lung cancer was significantly higher in frequent burners (more than 3 times [days] per week) than nonburners (adjusted OR = 3.78; 95% CI: 1.55-6.90). Subjects who seldom burned mosquito coils (less than 3 times per week) also had a significantly higher risk of lung cancer (adjusted OR = 2.67; 95% CI: 1.60-4.50). We further determined whether the frequency of burning mosquito coil was associated with different tumor types of lung cancer. The adjusted OR of adenocarcinoma increased with higher frequencies of burning mosquito coil, from 2.40 in infrequent burners to 3.51 in frequent burners (Table 2). ORs of the two higher categories were both statistically significant. A similar trend was seen when analysis was restricted to squamous cell carcinoma, but the 95% confidence intervals were wider.

**Table 2.** Adjusted odds ratios (OR) and 95% confidence intervals for lung cancer associated with exposure of Taiwanese to mosquito coil smoke.

Exposure status	Controls	All lung cancer patients		Adenocarcinoma patients		Squamous cell carcinoma patients	

	n=400	n=147	Adjusted OR	n=91	Adjusted OR	n=30	Adjusted OR
Frequency of burning							
> 3 times/week	26 ( 6.5%)	24 (16.3%)	3.78 (1.55-6.90)	16 (20.9%)	3.51 (1.48-8.32)	5 (20.0%)	4.67 (1.42-15.37)
≤ 3 times/week	45 (11.3%)	32 (21.8%)	2.67 (1.60-4.50)	18 (19.8%)	2.40 (1.35-4.26)	10 (33.3%)	4.64 (2.09-10.30)
None	329 (82.2%)	91 (61.9%)	1.00 (reference)	57 (62.6%)	1.00 (reference)	15 (46.7%)	1.00 ( reference )

* : Adjusted for age, marital status, occupation, active smoking, and tuberculosis history

95% confidence intervals in parentheses

Subsequently, we assessed whether frequency of mosquito coil use combined with tobacco smoking after adjustment for the effects of other confounders affected lung cancer development (Table 3). Nonsmokers who were nonburners were selected as the referent group (OR = 1). Nonsmokers who were infrequent burners had a 3.36-fold increased risk of lung cancer (95% CI: 1.76-6.40). The risk of lung cancer was also increased for nonsmokers who were frequent burners (more than 3 times/ week, OR = 3.89; 95% CI: 1.73-8.57), smokers who were nonburners (OR = 5.59; 95% CI: 2.34-13.36), smokers who were infrequent burners (OR = 11.84; 95% CI: 3.21-38.54), and smokers who were frequent burners (OR = 13.66; 95% CI: 3.64-51.29). Furthermore, the OR for the presence of both smoking and burning mosquito coil was greater than the sum of the OR for smoking and OR for burning mosquito coil. Synergy indices were greater than 1 (range, 1.58-1.69). These data clearly suggest that the effects of smoking and burning mosquito coil beyond additive.

**Table 3.** Risk of lung cancer associated with mosquito coil smoke exposure by cigarette smoking status.

Variables	Smoking status				
	
	Nonsmokers		Smokers		
	
	CA/CN^*^	OR (95% CI)^†^	CA/CN^*^	OR (95% CI)^†^	Synergy Index (95% CI)
Frequency of mosquito coil burning (times/week)
> 3	13/18	3.89 (1.73-8.57)	11/8	13.66 (3.64-51.29)	1.69 (1.21-2.36)
≤ 3	22/37	3.36 (1.76-6.40)	10/8	11.84 (3.21-38.54)	1.58 (1.12-1.87)
None	44/238	1.00 (reference)	47/91	5.59 (2.34-13.36)	

* : No. cases/no. controls

† : Data were calculated using unconditional logistic regression, and adjusted for age, marital status, occupation, and tuberculosis history

## DISCUSSION

Several environmental pollutants, such tobacco smoking,^25^ radon,^7^ and asbestos,^8^ have been linked to increased risk of lung cancer in humans. The evidence also suggests that in industrialized countries, outdoor air contaminants and indoor radon exposure are the two important causes of pollution-related lung cancer.^26^ In other regions of the world, however, other circumstances of exposure to pollutants, such as drinking water contaminated with arsenic^27^ and cooking practices^5^^,^^6^ may result in lung cancer. We further observed that exposure to MCS elevates risk for lung cancer in Taiwan, in the presence or absence of cigarette smoking.

Mosquito coils were used by consumers for protection against mosquitoes, whether to prevent disease transmission or simply to repel an annoying pest. In sub-tropical Taiwan, burning mosquito repellents is a common practice in low socio-economic, rural areas. In general, mosquito coils are usually used overnight in a bedroom for at least several months every year, especially during the summer. The burning practices of our subjects were similar, thus they have not been surveyed in our study. Epidemiological studies showed that long-term exposure to MCS might induce asthma and persistent wheeze in children.^14^^,^^15^ A case-control study also implicated MCS as a possible cause of nasopharyngeal carcinoma.^28^ Although only very limited published state-of-the-art inhalation toxicity studies are available to judge the potential health impact associated with exposure to MCS, there is a theoretical basis for our present findings. As mentioned previously, when a mosquito coil is burned, the insecticide evaporates with the smoke. The combustion of the remaining materials generates large amounts of submicrometer particles and gaseous pollutants. These particles may reach and coat the lower respiratory tract along with a wide range of organic compounds including polycyclic aromatic hydrocarbons. Moreover, burning mosquito coil can release a large amount of particulate matter and formaldehyde.^12^ Importantly, the association of polycyclic aromatic hydrocarbons,^29^ fine particles,^30^ and formaldehyde^31^ with human lung cancer has been suggested. Notably also, trace metals including cadmium and chromate contained in mosquito coil can be released during combustion.^32^ Interestingly, our recent study also observed carcinogenic chromate accumulation in lung tumor tissues of Taiwanese patients.^33^ Mosquito coils also contain as a synergist or active ingredient, octachlorodipropyl ether (S-2),^16^ a precursor of the potent lung carcinogen, bis(chloromethyl)ether.^18^ Das et al^34^ found higher frequency of chromosome aberrations and micronuclei in the pulmonary alveolar macrophages of rats that inhaled MCS over a long period. Thus, our results provide preliminary epidemiological evidence that MCS may have an important role in the development of lung cancer in Taiwan.

With regard to lung cancer histology and household exposure to MCS, our results indicated a similarly increased risk for the main histologic types of lung cancer, adenocarcinoma and squamous cell carcinoma (Table 2). The etiology of lung cancer is usually different by histologic type. However, on the basis of current data, we hypothesized that exposure to MCS might give rise to a similar mechanism of cancer development regardless of histologic type. However, we could not exclude the possibility that the size of squamous cell carcinoma sample in our study was insufficient to detect the expected association between tumor-type and mosquito coil use. Further exploration of the mechanism of MCS-induced lung cancer is needed.

Epidemiologic studies have demonstrated that cigarette smoking is the most important risk factor for lung cancer.^25^ Clearly, because lung cancers do not occur exclusively in smokers and the vast majority of smokers do not develop lung cancer, other etiological factors can independently (in the absence of smoking) or jointly (in the presence of smoking) cause lung cancer. In our study, the lung cancer risk of smokers with the highest exposure to MCS could be as high as 14-fold that of nonsmokers with no MCS exposure. The synergy indices ranged from 1.58 to 1.69, indicating a synergistic effect. This implies that chemicals within cigarette smoke and MCS might undergo the same metabolic transformation to become genotoxic metabolites that interact with DNA. The effects (DNA damage, mutations) of exposure to compounds with carcinogenic potential in cigarette smoke might be enhanced by MCS exposure, and MCS exposure might also affect the detoxification and clearance of cigarette smoke derivatives at the cellular level. Again, more studies are needed to make this determination.

Our study also revealed increased risk of lung cancer in individuals who are single, work in hazardous industrial environments, and have a history of tuberculosis. Single people tend to follow less healthy lifestyles, especially in relation to smoking^35^ and may lack the spiritual and social support of marriage. They thus may be less motivated to seek medical help and screening.^36^ Occupational exposure to carcinogens has also been suggested as a risk factor that could independently cause lung cancer.^37^^,^^38^ Our findings suggested an increased risk of lung cancer among individuals in occupations with hazardous exposures to suspected carcinogens. However, occupations were split into four categories (administrative worker, hazardous industry worker, farmer, and housewife). These categories may be incomplete or inaccurate, leading to occupation misclassification. Associations between nonmalignant respiratory disease, most notably tuberculosis, and subsequent lung cancer have been reported.^39^^,^^40^ A statistical relationship between preexisting tuberculosis infection and lung cancer was also observed in our study. A few mechanisms have been proposed for such an association. A chronic inflammatory process in the lung could enhance the effects of other carcinogenic exposures and stimulate cell proliferation and growth.^40^ Alternatively, a compromised immune response may increase susceptibility, or lung cancer may evolve directly from scar lesions.^41^^,^^42^

This case-control study has several limitations. The male-to-female ratio of lung cancer in Taiwanese is approximately at 2:1.^43^ However, the proportion of females with lung cancer in our present study was 45.6%, which seems to be relatively higher than in the general population. Female in our study were expected to consent to medical treatment. Furthermore, the mean age of our lung cancer patients at recruitment was younger for females than males (60.7 years vs. 66.3 years, respectively; p<0.001). Adenocarcinoma was the most common type of lung carcinoma in both sexs, but females (n=52 patients; 57.1%) had a significantly higher percentage of adenocarcinoma compared with males (n=39 patients; 42.9%; p<0.001). These suggest that our study had a larger proportion of patients with lung adenocarcinoma. However, this is unlikely to be a source of bias since there was no significant difference in sex distribution between cases and controls when we investigated the association between MCS exposure and lung cancer.

Because we interviewed subjects retrospectively, recall bias is possible. In our study, we collected information on frequency of mosquito coil burning but not on dose of MCS owing to the lack of environmental monitoring data. The available historical exposure data was too sparse and lacking in detail for a quantitative estimation of cumulative exposure level. Though subjects may have changed their MCS exposure pattern over time, we assumed that the exposure pattern was similar both during the reference and promotional periods of lung cancer. However, our control subjects were blinded to their classification status and were unaware of the hypotheses under study. Data pertaining to individual exposure was obtained without knowledge of health outcome. Consequently, exposure misclassification is assumed to be non-differential and, if existent, directed toward an underestimation of the risk for lung cancer. In addition, since subjects did not know the hypothesis of the present study, recall bias, if existent, should have been limited and should not have influenced our conclusions. Our controls were recruited from the Ophthalmology and Family Medicine Departments in the medical centers. Possibly, some controls with eye problems might have tried to avoid eye irritation from MCS and some controls with good health consciousness might have avoided indoor pollutant exposure. In addition, based on a survey performed in southern Taiwan by Yang et al,^11^ the prevalence of families that burn mosquito coils in the Taiwanese population is approximately 45%. However, the prevalence (18.5%) of exposure to MCS was smaller in our controls than the general population of Taiwan, possibly indicating that the MCS exposure of our controls is not representative of the general population. This might be resulted from a higher proportion of younger subjects in our controls, who live in urban areas and burn mosquito smoke infrequently as compared to elder rural inhabitants in Taiwan. Such result might over-estimate the magnitude of lung cancer risk of MCS use. Selection bias could have also resulted from the low participation rate of our controls. Lastly, the small sample size may have limited the statistical power of our study to detect a small increase in risk.

In conclusion, this study suggests that higher frequency of burning mosquito coils increases risk of lung cancer, and exposure to MCS increases the lung cancer risk of cigarette smokers. The associations between MCS and lung cancer development should be examined again in a large population-based sample, as the number of lung cancer cases was insufficient in this study.
